# Role of Cytokine Signaling during Nervous System Development

**DOI:** 10.3390/ijms140713931

**Published:** 2013-07-04

**Authors:** Alyaa Mousa, Moiz Bakhiet

**Affiliations:** 1 Department of Anatomy, Faculty of Medicine, Health Sciences Centre, Kuwait University, Safat 13060, Kuwait; E-Mail: alyaamousa@yahoo.com; 2 Department of Molecular Medicine, Princess Al-Jawhara Center for Genetics and Inherited Diseases, College of Medicine and Medical Sciences, Arabian Gulf University, P.O. Box 26671 Manama, Bahrain

**Keywords:** brain, immunity, interleukins, growth regulation

## Abstract

Cytokines are signaling proteins that were first characterized as components of the immune response, but have been found to have pleiotropic effects in diverse aspects of body function in health and disease. They are secreted by numerous cells and are used extensively in intercellular communications to produce different activities, including intricate processes engaged in the ontogenetic development of the brain. This review discusses factors involved in brain growth regulation and recent findings exploring cytokine signaling pathways during development of the central nervous system. In view of existing data suggesting roles for neurotropic cytokines in promoting brain growth and repair, these molecules and their signaling pathways might become targets for therapeutic intervention in neurodegenerative processes due to diseases, toxicity, or trauma.

## 1. Introduction

The nervous system is a system that engenders itself generation of its diverse components from a single progenitor, creating a dynamic self-regulating system. It is a complex network of nerves and cells that carry messages to and from the brain and spinal cord to various parts of the body. The central nervous system (CNS) used to be considered as an immune-privileged organ due to several facts, such as lacking a lymphatic system, possessing a blood brain barrier (BBB), and having a very weak constitutive expression of MHC antigens required for antigen presentation and tissue rejection. However, this is not the case anymore in view of the demonstrated ability of immune cells to cross the BBB among activation and that resident brain cells can generate immune response [1], in addition to the fact that immune signaling molecules were found to be existing early during brain development and contribute to the process of growth development [2]. A number of studies published in recent years have begun to shed some light on the evolutionary origins of the nervous system and its interactions with immunoregulatory molecules. These studies provide clues to some of the earliest stages in the development of the human brain.

## 2. Embryological Development of the Nervous System

Embryonic stem cells (ESCs) are pluripotent, not limited to particular cell fate and have the capacity to differentiate into any cell type. During development, before the formation of the neural stem cell niches, the cells of the inner cell mass generate the three germinal layers of embryos namely the endoderm, mesoderm, and ectoderm and will then give rise to tissue restricted stem cells [3]. During the third week of human gestation, the notochord, which is a cellular rod that defines the primitive axis of the embryo, stimulates the overlying ectoderm, inducing it to become neuroectoderm. This results in a strip of neuronal stem cells that runs along the back of the fetus. The entire nervous system is of neuroectodermal origin and its first rudiment is seen in the neural groove, which extends along the dorsal aspect of the embryo. By the end of the third week of gestation in humans, the elevation and ultimate fusion of the neural folds, the groove is converted into the neural tube near the anterior end of the notochord. Late in the fourth week, the anterior end of the neural tube becomes expanded to form the three primary brain-vesicles; the prosencephalon (forebrain), mesencephalon (midbrain), and rhombencephalon (hindbrain). The lower part of the neural tube forms the spinal cord. The cavity of the tube is then modified to form the brain ventricles and the central canal of the medulla spinalis. In the fifth week, the prosencephalon expands to form the cerebral hemispheres (the telencephalon) and the diencephalon. The rhombencephalon expands to form metencephalon (pons) and myelencephalon (medulla oblongata). The pons and the cerebellum form in the upper part of the rhombencephalon, whilst the medulla oblongata forms in the lower part. During the first month of gestation in humans, the CNS begins to form with the neurogenesis and migration of cells in the forebrain, midbrain, and hindbrain. There follows a sequence of developmental processes including proliferation, migration, differentiation, synaptogenesis, gliogenesis, myelination, and apoptosis [4]. Neurons are generated early along the central canal in the neural tube. The adult stem cells in mammalian brain are usually found in the subventricular zone (SVZ) in the lateral wall of the lateral ventricles. They are derived from the SVZ of the embryonic ganglionic eminences, and they give rise to neocortex, hippocampus, and olfactory bulb interneurons [5,6]. Neurons of the cerebral cortex are generated in the ventricular zone (VZ) of the neural tube, an epithelial layer of stem cells that lines the lateral ventricles. Glial and neuronal cells are developed from VZ “progenitor” cells. The firstly produced glial fibers are radially oriented, straddling the thickness of the cortex from the ventricular zone to the outer pial surface to give an outwards direction for neurons migration. Neurons on the cortical plate are converted into well-defined layers. Their processes, or axons, grow long distances to find and connect with appropriate partners, forming elaborate and specific circuits.

### 2.1. Progenitor Cell Proliferation

**Figure 1:**
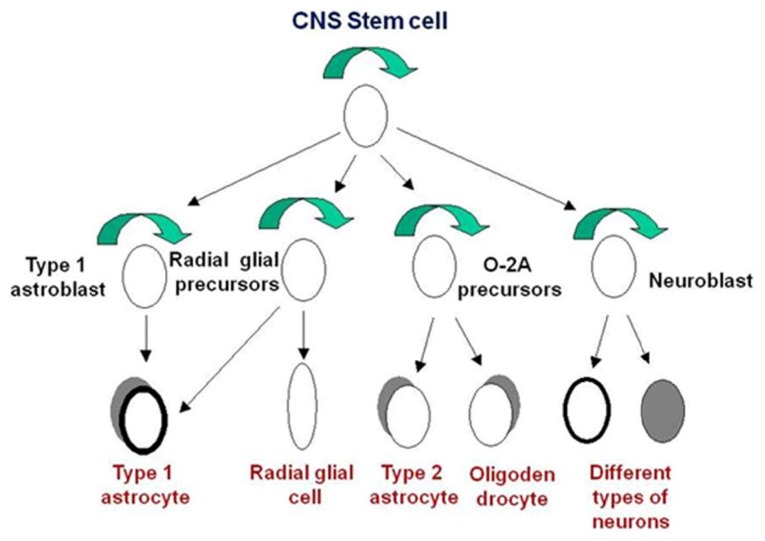
A diagram showing the relationships between the major central nervous system (CNS); cell types 
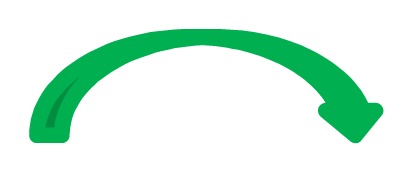
 Self- renewal.

A stem cell has the ability of self-renewal: making identical copies of itself, pluripotency and the ability to generate/regenerate tissues. Neuroblasts proliferate vigorously in the developing human CNS during the first trimester, and some neuronal populations do not cease to proliferate until the postnatal period. Extensive studies have revealed the presence of neural stem cells in embryonic and adult tissues [7]. Neural stem cells that can give rise to various types of neurons and glia may play a major role in mammalian CNS development and continue to function throughout adulthood (for review, see [8–10]). Primary cultures derived from mechanically dissociated specimens of first trimester human forebrain contain a heterogeneous cell population. These cells survive long term *in vitro* and the majority of cells in these cultures are glia (Figure 1).

Neurons and macroglia (astrocytes and oligodendrocytes) arise from precursors in the germinal layers of the developing brain, the VZ, and SVZ, respectively. Multipotential neural stem cells are present in these regions during development [11–14]. It is known that, during brain development, neurogenesis occurs before gliogenesis. Neurogenesis and gliogenesis are a continuous process and thus, multipotent stem cells can be found in the adult brain [reviewed in [13,15,16]. Furthermore, glial cells precursors and neurons have also been described [17–21]. For neurons and microglia, it has been suggested that their lineages are progressively segregated during development [22]. Thus, multipotential stem cells are only present early during brain development.

Regulation of neural stem cell self-renewal and differentiation is mediated through the combination of cell intrinsic factors and extracellular signaling molecules. The cell-intrinsic regulators coordinate with extrinsic signals to control the balance between neural stem cell self-renewal and differentiation.

*In vivo* first trimester human forebrain primary cultures express MHC antigen upon IFN-γ stimulation and higher cell survival [23]. Spontaneous pro-inflammatory and anti-inflammatory cytokine expression with age in human embryonic forebrain cells suggest that cytokines might be involved as a regulating factor promoting CNS development. [24]. Ultraviolet light-inactivated MV strongly induced expression of β-family mRNA in human first trimester astrocytes, especially MPC-1 and MIP-1β, suggesting a possible role of β-family chemokines in the pathogenesis of CNS inflammatory diseases [25]. Astrocytes and neurons in human first trimester forebrain cultures induce α and β chemokines indicating a role for cytokine/chemokine networks involvement in brain development [2]. RANTES (CCL5) was expressed preferentially in human fetal astrocytes in an age-dependent manner. RANTES induced tyrosine phosphorylation of several cellular proteins and nuclear translocation of STAT-1 in astrocytes [2]. During fetal development, the cells of the choroid plexus begin to secrete many small molecules that include leukemia inhibitory factor (LIF) and ciliary neurotrophic factor (CNTF) that signal through the Leukemia inhibitory factor receptor (LIFR). LIF/CNTF creates a gradient within the germinal zones that establishes the distinct layers of VZ and SVZ, and sustains self-renewal of the neural progenitors (NPs) [26]. The generated neurons are found in the SVZ of the lateral wall of the lateral ventricles. This layer is separated from the lateral ventricle by a layer of epithelial cells known as ependymal cells. Neuroblasts of the SVZ migrate as a network oriented chains [27,28] that converge on the rostral migratory stream (RMS) to reach the olfactory bulb, where they differentiate into local interneurons [27,29,30]. Type A migrating neuroblasts within the SVZ proceed as chains through tubes defined by the processes of slowly proliferating SVZ type B astrocytes. Type C cells, which are clusters of rapidly dividing immature precursors, are scattered along the network of migrating chains [31]. Isolation and molecular manipulation of the *in vivo* stem cells may be useful in the process of brain repair. It was also possible to cultivate adult SVZ neural stem cells, which have the capacity to self-renew and differentiate into neurons and glia [32,33]. They grow as spherical floating clusters (neurospheres) in the presence of epidermal growth factor (EGF) or basic fibroblast growth factor (bFGF) [34,35]. It has been suggested that the SVZ neural stem cells correspond to a rare population of relatively quiescent cells [32], while some recent studies suggested that ciliated ependymal cells correspond to the neural stem cells.

### 2.2. Neural Stem Cells in the Developing Brain

Early during development there are very few Glial fibrillary acidic protein (GFAP) positive cells within the brain and that neural stem cells at that time are different from those occurring in the adult brain [36]. It is possible to culture neuronal stem cells from any embryonic stage, giving rise to more neurons than those cultured at later periods [37]. This requires manipulation of the culture as, for example, deletion of the tumor suppressor gene, PTEN, allows neural stem cells derived from embryonic mouse cortex to retain their neurogenic capacity for longer periods in culture [38,39].

### 2.3. Cell Migration, Neuronal and Glial Cellular Interaction and Differentiation

Neuronal migration is, along with axon guidance, one of the fundamental mechanisms underlying the wiring of the brain. During development, some of the cells stop dividing and differentiate gradually into neurons and glial cells, which are the main cellular components of the brain. The newly generated neurons migrate as they travel from their sites of origin in the ventricular zone out to different parts of the developing brain by radial migration or tangential migration [40] to self-organize into different brain structures. Glial-guided migration seems to be the primary mechanism by which cortical neurons achieve their final destination [41,42]. Although radial patterns of neuronal migration have been thought to be essential for patterning these areas, direct observation of migrating cells in cortical brain slices has revealed that cells follow both radial and nonradial pathways [42]. Two general modes of migration have previously been defined in the developing nervous system: locomotion and nuclear translocation [43]. Locomotion involves movement of the entire cell, including its leading and trailing processes, often over considerable distances. The glial-guided radial movement of neurons is a well-defined example of cell locomotion. Migration toward the pial surface appears to occur along radial glia, which extend long processes from the ventricular to the pial surfaces. In contrast, during nuclear translocation (also called nucleokinesis) [44], the cell first extends a leading process in the direction of migration, and then moves the nucleus through the elongated process to its destination. Nucleokinesis occurs in two steps. First, the centrosome, together with the Golgi apparatus, moves forward, leading to the formation of a cytoplasmic swelling in the leading process ahead of the nucleus [45–47]. The centrosome is accompanied by additional organelles, including the Golgi apparatus, mitochondria, and the rough endoplasmic reticulum. Second, the nucleus moves toward the swelling, following the centrosome. These two steps are repeated producing the typical saltatory movement of migrating neurons. Two motor proteins: myosin II and cytoplasmic dynein appear directly implicated in nucleokinesis [48], however the precise mechanisms driving the nuclear movement remain controversial. Once the neurons have reached their regional positions, they extend axons and dendrites, which allow them to communicate with other neurons via synapses. Synaptic communication between neurons leads to the establishment of functional neural circuits that mediate sensory and motor processing, and underlie behavior. Embryonic and adult NSCs are lineally related: they transform from neuroepithelial cells into radial glia, then into cells with astroglial characteristics.

## 3. Cytokines

The term “cytokine” has been used to refer to the immunomodulating agents, such as interleukins (ILs) and interferons (IFNs). Cytokines are small, cell-signaling protein molecules that were first characterized as components of the immune response, but have since been found to play a much broader part in diverse aspects of physiology. They are secreted by numerous cells and are a category of signaling molecules used extensively in intercellular communication. Cytokines can be classified as proteins, peptides, or glycoproteins; the term “cytokine” encompasses a large and diverse family of regulators produced throughout the body by cells of diverse embryological origin [49]. Cytokines might exert their effect in the central nervous system both directly and indirectly [50–53]. Direct action means that cytokines themselves are present in the brain, in and/or around the various neuronal cells, while secondary effects that are the result of cytokine action on other targets represent the indirect pathways. Functionally, cytokines have been classified as being either pro-inflammatory (Th1-type, stimulatory) or anti-inflammatory (Th2-type, inhibitory) depending on the final balance of their effects. The group of structurally and functionally related cytokines consisting of interleukin-6 (IL-6), IL-11, ciliary neurotrophic factor (CNTF), leukemia inhibitory factor (LIF), [54] cardiotrophin 1 (CT-1), neuropoietin and cardiotrophin-like cytokine (CLC; also known as novel neurotrophin 1 (NNT1)), B cell stimulating factor 3 (BSF3), oncostatin M (OSM), IL-27, and IL-31 have been classified as one group. These cytokines have been named differently according to various aspects such as the sharing of a receptor subunit (*i.e*., the Glycoprotein gp130 family) or its physiological roles (*i.e*., neuropoietic family, for its effects on hematopoietic and nervous system) and the IL-6 family (after its “founding member”). Members of the IL-6 cytokine family are key regulators of inflammatory and immunological responses, and include IL-6, IL-11, CNTF, LIF, CT-1, CLC, and OSM, [55]. Each cytokine has a matching cell-surface receptor. Cytokines and their receptors are expressed in tissues of the nervous system, and might derive from invading immune, or resident, cells. Cytokines and their receptors are constitutively expressed by and act on neurons in the central nervous system, in both its normal and its pathological state, but cytokine over expression in the brain is an important factor in the pathogenesis of neurotoxic and neurodegenerative disorders. The binding of cytokines to these receptors induces homo- or hetero-dimerization of receptors and triggers activation of intracellular signaling cascades then alter cell functions [56]. This may include the upregulation and/or downregulation of several genes and their transcription factors, resulting in the production of other cytokines, an increase in the number of surface receptors for other molecules, or the suppression of their own effect by feedback inhibition. They signal through a gp130 receptor complex that activates the Janus Kinase-Signal Transducer, Activator of Transcription (JAK-STAT) and Mitogen-Activated Protein Kinase (MAPK) Signal Transduction pathways. Cytokines and other products of the immune cells can modulate the action, growth, differentiation, and survival of neuronal cells, while the neurotransmitter and neuropeptide release play a pivotal role in influencing the immune response. This review evaluates neuropoietic cytokines intercellular communication events and their vital role in growth regulation of the nervous system during nervous system development.

**Figure 2:**
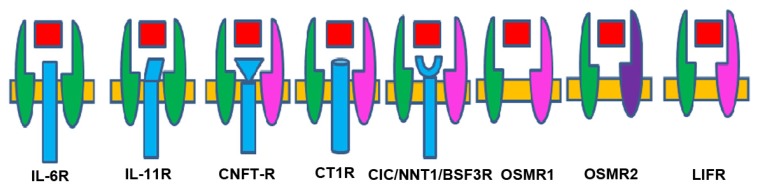
IL-6 family cytokines and their receptors Receptor complexes are composed of the signaling subunits gp 130 (green), LIF- R (pink) and OSM-R (lilac) and specific ligand binding receptors. (For IL-6, IL-11R, CNTF-R, CT-1R and CIC/NNT-1/BSF-3-R). IL-6 and IL-11 signal via a homodimer of gp 130 whereas ciliary neurotrophic factor (CNTF), cardiotrophin 1 (CT-1), and novel neurotrophin 1 (NNT-1) use a heterodimers of gp 130 and LIF-R. Leukemia inhibitory factor (LIF) and oncostatin M (OSM) do not use specific binding receptors. Binding of IL-6 cytokines will activate Janus Kinase-Signal Transducer, Activator of Transcription (JAK-STAT) and Mitogen-Activated Protein Kinase (MAPK) pathways.

Various combinations of receptor subunits and signaling pathways are used by different members of the neuropoietic cytokine family. Gp130 is a ubiquitously expressed, signal-transducing receptor that forms part of the receptor complex for several cytokines [57]. Gp130 controls the activity of a group of cytokines, namely, IL-6, IL-11, LIF, CNTF, CT-1, CLC, OSM, and NPN [58]. The cytokines that signal through the common receptor subunit gp130, including IL-6, LIF, CNTF, and OSM, have pleiotropic functions in CNS development [59]. Gp130 plays critical roles during development and gp130-deficient mice are embryonically lethal [60]. Receptors involved in recognition of the IL-6-type cytokines can be subdivided in the non-signaling α-receptors (IL-6Rα, IL- 11Rα, and CNTFRα) and the signal transducing receptors (gp130, LIFR, and OSMR). Two subunits, the LIF receptor and gp 130, are components of several receptors. Gp130 homodimers associate with specific interleukin receptors such as the IL-6 receptor (IL-6R) to mediate the actions of IL-6. IL-6, for example, binds first to its specific low affinity a-chain [61] and then to two subunits of gp130 to generate a high affinity complex [62]. IL-6 and IL-11 are the only IL-6-type cytokines that signal via gp130 homodimers. The remaining IL-6 type cytokines signal via heterodimers of, gp130, the LIFR, (LIF, CNTF, CT-1, and CLC) or gp130 and the OSMR (OSM) (Figure 2).

Human OSM has the exceptional capability to recruit two different receptor complexes. It forms both LIFR-gp130 and OSMR-gp130 heterodimers. LIF and OSM directly engage their signaling receptor subunits without requirement for additional α-receptor subunits [63,64]. IL-11R heterodimer such as IL-11Rα involved in ligand recognition, and gp130 involved in signal transduction. LIF binds to heterodimers of LIF receptor (LIFR) and gp130. CNTF initially binds to a specific α-chain [65] then to the LIF receptor and gp130 [66]. LIFR–gp130 heterodimers can also associate with other receptor subunits to bind CNTF and CT-1. The OSMR forms heterodimers with gp130 to bind OSM. IL-6-type cytokines exert their action via the signal transducers gp 130, LIF receptor and OSM receptor leading to the intracellular activation of the JAK/STAT and MAPK cascades [55]. IL-6 receptor has two components; the non-signaling α-receptors (IL-6Rα, IL-11Rα, and CNTFRα) and the signal transducing receptors (gp130, LIFR, and OSMR). The latter associate with JAKs and become tyrosine phosphorylated in response to cytokine stimulation.

In addition to the membrane-bound receptor, a soluble form of IL-6R (sIL-6R) has been purified from human serum and urine [67,68]. The sIL-6R family of cytokines (sIL-6R, sIL-11R, sCNTF-R) is agonist, capable of transmitting signals through interaction with the universal signal-transducing receptor for all IL-6 family cytokines, gp130. IL-6 with sIL-6R stimulate cells, which only express gp130 [69,70] in a process that is termed trans-signaling [69–71]. The sIL-6R enhances the effect of IL-6 stimulation, by binding IL-6 and bringing it to the membrane bound gp130 subunits, allowing them to transduce the signal into the cell. Embryonic stem [72,73], early hematopoietic progenitor cells [74–78], T cells [79–81], many neural cells [82,83], smooth muscle cells [84], mesothelial cells [85,86], and endothelial cells [87], among others, are only responsive to IL-6 in the presence of sIL-6R [71]. Although many neuronal cells are capable of producing IL-6, they remain unresponsive to stimulation by IL-6 itself. Differentiation and survival of neuronal cells can, however, be mediated through the action of sIL-6R. For example, sympathetic and sensory neurons from neonatal superior cervical ganglia, retinal ganglion cells and embryonic dorsal root ganglia show a marked increase in survival and neurite outgrowth when stimulated by the [sIL-6R/IL-6] complex [82,88,89]. In addition, sIL-6R may perform a role in axon growth from dorsal root ganglia, and in the development of Schwann cell progenitors, which express myelin basic protein after activation with a combination of IL-6 and sIL-6R [90]. Thus, the sIL-6R/IL-6 complex can stimulate neurites outgrowth, promoting survival of neurons, and hence may be important in nerve regeneration through the promotion of remyelination events. Increased and systemic expression of IL-6 with sIL-6Rα is less harmful to the brain than to other organs according to a study [91].

The neuropoietic cytokine family has been shown to alter neural stem cell (NSC) self-renewal and progenitor cell division and differentiation, which could be mediated by the JAK/STAT pathway.

### 3.1. Cytokines Signaling Pathways

During the last 10 years, a lot of advances have been made in illustrating how cytokines transmit signals via pathways using the JAK protein tyrosine kinases and the STAT proteins. Accumulating evidence indicates that the latent transcription factors, STAT, play an essential role in cytokine signaling pathways. The JAK family consists of four members (JAK1-3 and TYK2). The existence of JAK-family members in the nervous system was first reported by [92] who provided evidence of mRNA for JAK1 in the retina and in whole-brain extracts during development [92]. JAK2 was detected in various rat brain regions during embryonic and postnatal stages dividing and postmitotic cells [93]. Very low levels of JAK3 were found, while TYK2 was not detected [93]. The STAT protein family was discovered in the course of studies of signaling specificity from IFN receptors [94]. STAT proteins have been shown to play an essential role in cytokine-mediated biological responses. Several STAT family members have been identified, and the STAT family now consists of seven members (STAT1-4, STAT5a, STAT5b, and STAT6). STAT3 was first described as a DNA-binding activity from IL-6-stimulated hepatocytes, capable of selectively interacting with an enhancer element in the promoter of acute-phase genes, known as the acute-phase response element [95–98]. This same protein, a close relative of STAT1, is activated by the entire family of IL-6-type cytokines, which signal through gp130 and related receptors [57,63,99]. Fate of neural stem cells during development is regulated by cell-intrinsic programs, such as epigenetic modification (including DNA methylation), and signaling crosstalk of cell-external mediators (including IL-6 family cytokines) [100]. IL-6-type cytokines evoke a number of distinct responses in different cells, including induction of an acute-phase response in hepatoma cells, stimulation of proliferation in B lymphocytes, activation of terminal differentiation and growth arrest in monocytes [63], and maintenance of the pluripotency of embryonic stem cells [101–104]. Regulatory circuits of STAT3 and miRNAs play important roles in the neural lineage differentiation of ES cells. Neural stem cells differentiate into the three main neural lineages: neurons, astrocytes, and oligodendrocytes [105]. In the developing central nervous system, activation of STAT3 is known to direct the differentiation of neural stem cells toward astrocytes and to suppress neurogenesis [106]. STAT3-mediated signaling is one of the main mechanisms for promoting astrocyte differentiation by inhibiting neuronal differentiation in the embryonic cortex [107]. It has been known that STAT3 can promote NSC proliferation, while in the presence of activated BMP and Notch signaling, it induces astrogenesis [108,109]. The finding that STAT3 is involved in all these distinct functions has suggested that STAT3 is the major signal transducer downstream of gp130-like receptors. STAT3 is required for embryogenesis, and ablation of STAT3 led to early embryonic lethality [110]. In fact, loss of STAT3 is lethal even in embryonic stem cells [103,104]. Like STAT3, other components of the gp130 signaling system are also required for embryogenesis. For instance, loss of genes for various receptors, including gp130 and leukemia inhibitory factor receptor β (LIFRβ), leads to embryonic lethality [111]. IL-6-type cytokines use gp130 as a common receptor subunit. The binding of ligand to gp130 activates the JAK/STAT signal transduction pathway, where STAT3 plays a central role in transmitting the signals from the membrane to the nucleus. STAT3 is essential for gp130-mediated cell survival and G1 to S cell-cycle-transition signals. STAT3 mediating the cell growth, diferentiation and survival signals through the IL-6 family of cytokine receptors. JAK1, which appears to be the essential receptor-associated kinase for gp130 action [112,113], is also essential in promoting biologic responses induced by a select subset of cytokine receptors [114]. Tyrosine phosphorylation, in response to cytokine stimulation such as IL-6, CNTF, LIF, and OSM, [115] is mediated by a Janus kinase, most often JAK1 [112], and is required for STAT3 dimerization and move into the nucleus, recruiting p300 and binding to specific sequences in target gene promoter [115] (Table 1).

### 3.2. Cytokines Signaling Pathway in the Nervous System

Cytokines play a central role in maintaining self-renewal in mouse embryonic stem (ES) cells through a member of the IL-6 type cytokine family termed LIF. The members of this cytokine family have pro- as well as anti-inflammatory properties and are major players in haematopoiesis, as well as in acute-phase and immune responses of the organism. They activate target genes involved in differentiation, survival, apoptosis, and proliferation. The IL-6 cytokines are critical for fetal neurodevelopment, and participate in CNS neurodegenerative diseases [131,132]. IL-6 cytokine family signaling occurs when the cytokine binds to its cognate receptor, causing it to associate with gp130, the common signal transducing subunit for IL-6 cytokines. IL-6-type cytokines bind to plasma membrane receptor complexes containing the common signal transducing receptor chain gp130. Signal transduction involves the activation of JAK tyrosine kinase family members, leading to the activation of transcription factors of the STAT family. These cytokines bind to class I cytokine receptors, membrane proteins with a characteristic modular architecture that do not have intrinsic enzymatic activity, and for signaling often need to recruit additional receptor proteins shared by different cytokines: gp130, βc, or γc. The IL-6 family of cytokines recruits gp130 for signaling. The sharing of gp130 explains at least in part the redundancy of the actions of these cytokines. The cytokines IL-6 and IL-11 bind to their corresponding receptors IL-6R and IL-11R, respectively, resulting in complex formations, which associate with gp130, leading to gp130-homodimer formation and signal initiation. LIF, CNTF, OSM, CT-1, NPN, and NNT-1, signal via gp130/LIF-R heterodimeric receptor complexes [57]. OSM signals via a receptor complex consisting of gp130 and OSM-R. IL-27 has been shown to act via a gp130/WSX-1 heterodimeric receptor complex [133]. However, IL-31 is the only IL-6 type cytokine that does not require the receptor chain gp130 but instead induces the formation of a heterodimer of gp130-like receptor (GPL) together with the OSM-R. Another major signaling pathway for IL-6-type cytokines is the MAPK cascade. Recent reviews on the subject of signal transduction via the JAK/STAT pathway have been published [134–136]. Ligand-receptor complex formation gives rise to gp130-associated JAKs activation, which recruits and phosphorylates STAT proteins, mainly STAT3. Phosphorylated STAT3 dimerizes and translocates into the nucleus where it induces transcription of target genes such as Bcl-xL and Bcl-2, which are critical for promoting neuronal survival [137]. Astrocytes and microglia are the major source of IL-6 and IL-6 members in the CNS [138,139], a process which requires stimulatory effect by different factors such as cytokines, PGE2 and neurotransmitters [138,139]. Neurons can also bind these cytokines and initiate signaling via expression of gp130 and ligand-specific receptors [140,141]. STAT3 besides self-renewal might also prevent apoptosis in a proB-cell line and in T cells [142,143], and constitutive activation of STAT3 blocks apoptotic processes in myeloma cells [144]. Members of the IL-6 family include IL-6, IL-11, LIF, CNTF, CT-1, NNT-1, OSM, NPN, IL-27, and IL-31 with the exception of IL-31, all IL-6 type cytokines share the membrane glycoprotein gp130 as a common receptor and signal transducer subunit (reviewed in references [55,145]. Viral IL-6 (vIL-6) from the human herpes virus 8 (HHV-8) also signals via a gp130 homodimer, but without the need of the αIL-6R [146]. Other IL-6 type cytokines need additional specific α receptors, including the glycosylphosphatidylinositol (GPI)-anchored CNTF-R for CNTF, CLC, NPN, and NNT-1, as well as the soluble Epstein-Barr-virus induced Gene 3 (EBI-3) for p28 (IL-27). CT-1 has been described to act directly via a gp130/LIF-R heterodimer [55,147].

It is becoming increasingly evident that the neurotrophic effects of LIF involve the complex interaction of different signal transduction pathways and the regulation of gene expression. LIF activates the JAK-STAT3 pathway through the class I cytokine receptor gp130 belongs to the IL-6 receptor family [148], which forms a trimeric complex with LIF and the class I cytokine receptor LIF receptor b. The LIF receptor is a class I cytokine receptor that and is a heterodimer composed of the common glycoprotein gp130 subunit and the LIFβ receptor subunit. In addition to LIF, the gp130/LIFβ receptor complex is involved in mediating the actions of other neuropoietic cytokines, including ciliary neurotrophic factor (CNTF), cardiotrophin-1, and oncostatin M, and this commonality underlies the functional overlap and apparent redundancy shown by these peptides [148,149]. Upon binding to its heterodimeric receptor formed by gp130 (IL6ST) and LIFR, LIF elicits several downstream signaling events mainly transmitted to the nucleus through JAK-STAT3, MEK (MAPK/ERK kinase), and PI3K (phosphoinositide 3-kinases) pathways [150]. These pathways are primarily associated with cell survival, differentiation, and regulation of apoptosis, respectively, in non-neural stem cells, such as mouse ESCs [151].

IL-6 is a prototypical four-helix bundle cytokine that is the founder member of the neuropoietic cytokines. IL-6 is a cytokine not only involved in inflammation and infection responses but also in the regulation of metabolic, regenerative, and neural processes. Activation of the STAT3 signaling pathway in neurons has been shown to promote neurite outgrowth and protect against neuronal death [132,140,152,153]. In addition, leptin-induced STAT3 activation inhibits glutamate-induced neuronal death *in vivo* and *in vitro*, [137]. These findings indicate that IL-6 cytokines contribute to neuroprotection, likely through STAT3 activation. For IL-6 specifically, a hexamer forms (two IL-6, two IL-6R and two gp130) that can activate intracellular tyrosin-kinases such as Janus kinase (JAK) and, to a lesser extent, TYK, which, in turn, activate a number of proteins including the STAT (signal transducer and activator of transcription) family of transcription factors, or the RAS-RAF-MAPK pathway, PI3K, or IRS (insulin receptor substrate) [154].

## 4. Cytokines in Central Nervous System (CNS) Development and Function

Cytokines play essential roles in health and disease as their well-known involvement in infection, pregnancy, and bone, muscle, and cardiovascular function, and these signaling proteins are central to many brain processes. Cytokines and their receptors are expressed physiologically in CNS cells and are important for development and function of the brain, *i.e.*, the neuropoietic cytokine family (IL-6, IL-11, CNTF, LIF, CT-1, CLC; also known as NNT1 or BSF3, OSM and IL-27). The members of this cytokine family have overlapping, pleiotropic effects on a variety of different cell types and activate target genes involved in survival, apoptosis, proliferation, and differentiation, as well as suppression of differentiation [55]. They have been identified as growth factors and key elements in gliogenesis [155]. These cytokines have recently been shown to have signaling functions in adult and the normal brain developing in particular in the regulation of neurogenesis and stem cell fate. These proteins have more functions, as key modulators of synaptic plasticity and of various behaviors [156], factors have trophic effects on subsets of neurons. Human CNTF showed the well-known trophic effect on both chick and rat DRG neurones. Human and murine LIF and, at unphysiologically high doses, human OSM were trophic for rat neurones, but failed to promote chick DRG cell survival. Human IL-11, murine IL-6, and human IL-6 did not improve chick or rat DRG neurone survival; soluble human IL-6 receptor alpha did not increase sensitivity to human IL-6. Thus, human CNTF as well as murine and human LIF had special neurotrophic properties compared with other related cytokines [54]. Mouse CT-1 showed prominent trophic effects that were comparable to those of CNTF and LIF. Soluble IL-11 receptor alpha even had slight neurotrophic effects by itself. These results suggest that CT-1 and IL-11 might also be involved in the physiological regulation of sensory neuron survival [157]. CNTF (gliogenic factor) exposure resulted in a higher number of cells expressing GFAP responding to ATP in retinal progenitor cultures [158]. CNTF-in transduced mice showed a significant increase in GAP43 expression in sensory neurons, a marker of axonal regeneration and improves regeneration of injured sciatic nerve [159]. Neuropoietic cytokines such as LIF are important for initiating GFAP expression but not astrocyte cell fate determination and that different cytokines induce differentiation into cells with specific morphologies and, presumably, functions [160].

### 4.1. Interleukin-6 (IL-6)

The biology of IL-6 is complex with pro- or anti-inflammatory cytokine or neurotrophic actions, depending on the signaling pathway activated review, [161]. IL-6 may act as a developmental neurotrophic factor [162,163], *in vitro* IL-6 clearly decreases the differentiation of neural stem/progenitor cells into neurons [164,165], and it has been shown to improve *in vitro* survival of several classes of neurons [166,167]. Moreover, it is suggested that IL-6 predominantly plays a protective role by improving survival of neurons in culture [168–170], protecting neurons from excitotoxic and ischemic insults [171–174], and promoting the growth of axons and consequently the number of synapses in a region [117,175–177]. Additionally, evidence shows that IL-6 may play a major role in the establishment, function, and modification of synaptic connections [178], and promoting synaptic plasticity, LTP, and memory consolidation [179,180]. Furthermore, IL-6 is found to regulate survival of differentiated neurons and the development of astrocytes [149,181]. In contrast, IL-6 is involved in oligodendrogliogenesis and astrogliogenesis [182,183]. However, other studies claimed that IL-6 promotes both gliogenesis (through the STAT-3 pathway) and neurogenesis (MAPK/CREB pathway) [184,185].

### 4.2. Interleukin-11 (IL-11)

This cytokine is a functionally pleiotropic cytokine [186,187] that was initially characterized because of its ability to stimulate the proliferation of an IL-6-dependent plasmacytoma cell line, T1165 [188]. Other biological actions of IL-11 are shared with IL-6, LIF, CNTF, and OSM [187,189–192] CT-1 and IL-11 might also be involved in the physiological regulation of sensory neuron survival [157]. These activities include the induction of multipotential haemopoietic progenitor cell proliferation.

### 4.3. Leukemia Inhibitory Factor (LIF)

Also known as cholinergic differentiation factor, are involved in these processes and may have an important neuroprotective role [148,149]. LIF and its receptor were expressed early in the development of the nervous system. It has been suggested that LIF, synthesized prenatally by neural progenitors, might act in an autocrine/paracrine manner [193]. In the rodent nervous system, LIF promotes regeneration of transected/axotomized nerves [194,195] and protects neurons during development [196] and in disease [197,198]. In mice, LIF is well known for its role during development in promoting totipotent embryonic stem cell (ESC) self-renewal. LIF is a cytokine known to influence proliferation and/or survival of mouse primordial germ cells (PGC) in culture. LIF promotes NSC self-renewal in the normal adult brain *in vivo* [199]. LIF signaling significantly promotes human neural progenitor (NP) cell proliferation, survival, and differentiation *in vitro* [200]. Indeed, LIF has been shown to enhance the generation of neurons in human CNS-derived stem cell cultures [201]. LIF is essential for subventricular zone neural stem cell and progenitor homeostasis [202]. In humans, LIF is required for the long-term growth of embryonic human neural stem cells (NSCs) [203,204]. In addition, LIF might alter human NSC differentiation, as it promotes neurogenesis in stem and progenitor cells derived from the adult human olfactory bulb [200,204]. LIF and CTNF induce premature generation of astrocytes *in vitro* through activation of the JAK-STAT and MAPK pathways [192,205]. Interestingly, LIF mediates astrogliogenesis in late (>E15), but not early (E12–E14), cortical progenitors in mice [105,192]. LIF is protective in trauma, but in response to neuronal input, was also recently shown to promote myelination in cortical/glial cocultures. The expression of LIF mRNA was upregulated in astroglia in response to neuronal activity [206]. A concomitant increase in myelination after electrical activation occurred in cocultures from wild-type mice, but not in those from LIF knockout mice. Furthermore, LIF-deficient mice exhibit fewer glial fibrillary acidic protein (GFAP)-positive astrocytes and lower levels of MBP [207].

### 4.4. Ciliary Neurotrophic Factor (CNTF)

CNTF promotes the survival and differentiation of developing motor neuron [208]. In culture, CNTF and LIF markedly stimulated neurite outgrowth of mature retinal ganglion cells (RGCs) [209]. Several molecules, including CNTF, that act through the LIF receptor or gp130 signaling chain have neuroprotective actions on either neurons or oligodendrocytes, and lead to enhancing oligodendrocyte survival in the face of inflammatory Cytotoxicity [210].

### 4.5. Neuropoietin (NP)

NP can play a role in the development of the nervous system, as well as affect adipogenesis and fat cell function. NP-induced activation of STAT3 tyrosine phosphorylation is independent of leukemia inhibitory factor receptor (LIFR) phosphorylation and degradation. Although it is widely accepted that NP signals via the LIFR, studies reveal that NP results in phosphorylation of gp130, but not LIFR [211]. CNTFR/LIFR/gp130-mediated signaling supports the maintenance of forebrain neural stem cells, likely by suppressing restriction to a glial progenitor cell fate [212]. These cytokines have been shown to alter neural stem cell (NSC) self-renewal and progenitor cell division and differentiation, which could be mediated by the Janus kinase-signal transducer and activator of transcription (JAK/STAT) pathway [160].

## 5. Conclusions

Neurotropic cytokines and their signaling pathways are critical factors involved in brain growth regulation and repair. Thus, they might become targets for future therapeutic interventions during neurodegenerative processes occurring as a result of diseases, toxicity, or due to trauma.

## Figures and Tables

**Table 1:** IL-6 Family cytokine demonstrated to act through the JAK-STAT pathway.

Ligand	STAT	References
IL6	STAT1, STAT3	[63,116–119]
IL-11	STAT1, STAT3	[116]
CNTF	STAT1, STAT3	[105,116,120–122]
LIF	STAT1, STAT3	[116,122]
CT-1	STAT1, STAT3	[116,123–125]
CLC (NNT1)	STAT1, STAT3	[126,127]
OSM	STAT1, STAT3	[116,128]
IL-27	STAT1, STAT3	[129]
IL-31	STAT1, STAT3	[130]

## References

[1] Ousman S.S., Kubes P. (2012). Immune surveillance in the central nervous system. Nat. Neurosci.

[2] Bakhiet M., Tjernlund A., Mousa A., Gad A., Strömblad S., Kuziel W.A., Seiger A., Andersson J. (2001). RANTES promotes growth and survival of human first-trimester forebrain astrocytes. Nat. Cell Biol.

[3] Thomson J.A., Itskovitz-Eldor J., Shapiro S.S., Waknitz M.A., Swiergiel J.J., Marshall V.S., Jones J.M. (1998). Embryonic stem cell lines derived from human blastocysts. Science.

[4] Herschkowitz N., Kagan J., Zilles K. (1997). Neurobiological bases of behavioral development in the first year. Neuropediatrics.

[5] Corbin J.G., Nery S., Fishell G. (2001). Telencephalic cells take a tangent: Non-radial migration in the mammalian forebrain. Nat. Neurosci.

[6] Marin O., Rubenstein J.L. (2001). A long, remarkable journey: Tangential migration in the telencephalon. Nat. Rev. Neurosci.

[7] Temple S. (2001). The development of neural stem cells. Nature.

[8] Alvarez-Buylla A., Temple S. (1998). Stem cells in the developing and adult nervous system. J. Neurobiol.

[9] Gage F.H. (2000). Mammalian neural stem cells. Science.

[10] Van der Kooy D., Weiss S. (2000). Why stem cells?. Science.

[11] Davis A.A., Temple S.A. (1994). Self-renewing multipotential stem cell in embryonic rat cerebral cortex. Nature.

[12] Johe K.K., Hazel T.G., Muller T., Dugich-Djordjevic M.M., McKay R.D. (1996). Single factors direct the differentiation of stem cells from the fetal and adult central nervous system. Genes Dev.

[13] Weiss S., Reynolds B.A., Vescovi A.L., Morshead C., Craig C.G., van der Kooy D. (1996). Is there a neural stem cell in the mammalian forebrain?. Trends Neurosci..

[14] Qian X., Goderie S.K., Shen Q., Stern J.H., Temple S. (1998). Intrinsic programs of patterned cell lineages in isolated vertebrate CNS ventricular zone cells. Development.

[15] Gage F.H., Ray J., Fisher L.J. (1995). Isolation, Characterization, and use of Stem Cells from the CNS. Annu. Rev. Neurosci.

[16] Temple S., Alvarez-Buylla A. (1999). Stem cells in the adult mammalian central nervous system. Curr. Opin. Neurobiol.

[17] Luskin M.B., Pearlman A.L., Sanes J.R. (1988). Cell lineage in the cerebral cortex of the mouse studied *in vivo* and *in vitro* with a recombinant retrovirus. Neuron.

[18] Price J., Thurlow L. (1988). Cell lineage in the rat cerebral cortex: A study using retroviral-mediated gene transfer. Development.

[19] Mayer-Proschel M., Kalyani A.J., Mujtaba T., Rao M.S. (1997). Isolation of lineage-restricted neuronal precursors from multipotent neuroepithelial stem cells. Neuron.

[20] Rao M.S., Noble M., Mayer-Proschel M. (1998). A tripotential glial precursor cell is present in the developing spinal cord. Proc. Natl. Acad. Sci. USA.

[21] Shi J., Marinovich A., Barres B.A. (1998). Purification and characterization of adult oligodendrocyte precursor cells from the rat optic nerve. J. Neurosci.

[22] Jacobson M. (1991). Developmental Neurobiology.

[23] Mousa A., Mustafa M., Kjaeldgaard A., Wahlberg L., Seiger A., Bakhiet M. (1998). Interactions between human embryonic forebrain cells and the cytokines interferon-gamma and interleukin-4. Int. J. Mol. Med.

[24] Mousa A., Seiger A., Kjaeldgaard A., Bakhiet M. (1999). Human first trimester forebrain cells express genes for inflammatory and anti-inflammatory cytokines. Cytokine.

[25] Xiao B.G., Mousa A., Kivisäkk P., Seiger A., Bakhiet M., Link H. (1998). Induction of beta-family chemokines mRNA in human embryonic astrocytes by inflammatory cytokines and measles virus protein. J. Neurocytol.

[26] Gregg C., Weiss S. (2005). CNTF/lif/gp130 receptor complex signaling maintains a VZ precursor differentiation gradient in the developing ventral forebrain. Development.

[27] Doetsch F., Alvarez-Buylla A. (1996). Network of tangential pathways for neuronal migration in adult mammalian brain. Proc. Natl. Acad. Sci. USA.

[28] Lois C., Garcia-Verdugo J.M., Alvarez-Buylla A. (1996). Chain migration of neuronal precursors. Science.

[29] Luskin M.B. (1993). Restricted proliferation and migration of postnatally generated neurons derived from the forebrain subventricular zone. Neuron.

[30] Lois C., Alvarez-Buylla A. (1994). Long-distance neuronal migration in the adult mammalian brain. Science.

[31] Doetsch F., Garcia-Verdugo J.M., Alvarez-Buylla A. (1997). Cellular composition and three-dimensional organization of the sub-ventricular germinal zone in the adult mammalian brain. J. Neurosci.

[32] Morshead C.M., Reynolds B.A., Craig C.G., McBurney M.W., Staines W.A., Morassutti D., Weiss S., van der Kooy D. (1994). Neural stem cells in the adult mammalian forebraina relatively quiescent subpopulation of subependymal cells. Neuron.

[33] Gritti A., Frolichsthal-Schoeller P., Galli R., Parati E.A., Cova L., Pagano S.F., Bjornson C.R., Vescovi A.L. (1999). Epidermal and fibroblast growth factors behave as mitogenic regulators for a single multipotent stem cell-like population from the subventricular region of the adult mouse forebrain. J. Neurosci.

[34] Reynolds B., Weiss S. (1992). Generation of neurons and astrocytes from isolated cells of the adult mammalian central nervous system. Science.

[35] Gritti A., Cova L., Parati E.A., Galli R., Vescovi A.L. (1995). Basic fibroblast growth factor supports the proliferation of epidermal growth factor-generated neuronal precursor cells of the adult mouse CNS. Neurosci. Lett.

[36] Fox I.J., Paucar A.A., Nakano I., Mottahedeh J., Dougherty J.D., Kornblum H.I. (2004). Developmental expression of glial fibrillary acidic protein mRNA in mouse forebrain germinal zones-implications for stem cell biology. Brain Res. Dev. Brain Res.

[37] Qian X., Shen Q., Goderie S.K., He W., Capela A., Davis A.A., Temple S. (2000). Timing of CNS cell generation: A programmed sequence of neuron and glial cell production from isolated murine cortical stem cells. Neuron.

[38] Groszer M., Erickson R., Scripture-Adams D.D., Dougherty J.D., Le Belle J., Zack J.A., Geschwind D.H., Liu X., Kornblum H.I., Wu H. (2006). PTEN negatively regulates neural stem cell self-renewal by modulating G0–G1 cell cycle entry. Proc. Natl. Acad. Sci. USA.

[39] Groszer M., Erickson R., Scripture-Adams D.D., Lesche R., Trumpp A., Zack J.A., Kornblum H.I., Liu X., Wu H. (2001). Negative regulation of neural stem/progenitor cell proliferation by the PTEN tumor suppressor gene *in vivo*. Science.

[40] Nadarajah B., Brunstrom J.E., Grutzendler J., Wong R.O., Pearlman A.L. (2001). Two modes of radial migration in early development of the cerebral cortex. Nat. Neurosci.

[41] Rakic P. (1972). Mode of cell migration to the superficial layers of fetal monkey neocortex. J. Comp. Neurol.

[42] O’Rourke N.A., Dailey M.E., Smith S.J., McConnell S.K. (1992). Diverse migratory pathways in the developing cerebral cortex. Science.

[43] Book K.J., Morest D.K. (1990). Migration of neuroblasts by perikaryal translocation: Role of cellular elongation and axonal outgrowth in the acoustic nuclei of the chick embryo medulla. J. Comp. Neurol.

[44] Morris N.R., Efimov V.P., Xiang X. (1998). Nuclear migration, nucleokinesis and lissencephaly. Trends Cell Biol.

[45] Bellion A., Baudoin J.P., Alvarez C., Bornens M., Metin C. (2005). Nucleokinesis in tangentially migrating neurons comprises two alternating phases: Forward migration of the Golgi/centrosome associated with centrosome splitting and myosin contraction at the rear. J. Neurosci.

[46] Schaar B.T., McConnell S.K. (2005). Cytoskeletal coordination during neuronal migration. Proc. Natl. Acad. Sci. USA.

[47] Higginbotham H.R., Gleeson J.G. (2007). The centrosome in neuronal development. Trends Neurosci.

[48] Vallee R.B., Seale G.E., Tsai J.W. (2009). Emerging roles for myosin II and cytoplasmic dynein in migrating neurons and growth cones. Trends Cell Biol.

[49] Gilman A., Goodman L.S., Hardman J.G., Limbird L.E. (2001). Pharmacological basis of therapeutics.

[50] Besedovsky H.O., del Rey A., Klusman I., Furukawa H., Monge Arditi G., Kabiersch A. (1991). Cytokines as modulators of the hypothalamus-pituitary-adrenal axis. J. Steroid Biochem. Mol. Biol.

[51] Breder C.D., Dinarello C.A., Saper C.B. (1988). Interleukin-1 immunoreactive innervation of the human hypothalamus. Science.

[52] Plata-Salaman C.R., Oomura Y., Kai Y. (1988). Tumor necrosis factor and interleukin-1 betaSuppression of food intake by direct action in the central nervous system. Brain Res.

[53] Sternberg E.M. (1997). Neural-immune interactions in health and disease. J. Clin. Invest.

[54] Simon R., Their M., Kruttgen A., Rose-John S., Weiergraber O., Heinrich P.C., Schroder J.M., Weis J. (1995). Human CNTF and related cytokines: Effects on DRG neurone survival. Neuroreport.

[55] Heinrich P.C., Behrmann I., Haan S., Hermanns H.M., Muller-Newen G., Schaper F. (2003). Principles of interleukin (IL)-6-type cytokine signalling and its regulation. Biochem. J.

[56] Kishimoto T., Taga T., Akira S. (1994). Cytokine signal transduction. Cell.

[57] Taga T., Kishimoto T. (1997). Gp130 and the interleukin-6 family of cytokines. Annu. Rev. Immunol.

[58] Fasnacht N., Muller W. (2008). Conditional gp130 deficient mouse mutants. Semin. Cell Dev. Biol.

[59] Shimazaki T., Shingo T., Weiss S. (2001). The ciliary neurotrophic factor/leukemia inhibitory factor/gp130 receptor complex operates in the maintenance of mammalian forebrain neural stem cells. J. Neurosci.

[60] Silver J.S., Hunter C.A. (2010). gp130 at the nexus of inflammation, autoimmunity, and cancer. J. Leukoc. Biol..

[61] Yamasaki K., Taga T., Hirata Y., Yawata H., Kawanishi Y., Seed B., Taniguchi T., Hirano T., Kishimoto T. (1988). Cloning and expression of the human interleukin-6 (BSF-2/IFN beta 2) receptor. Science.

[62] Hibi M., Murakami M., Saito M., Hirano T., Taga T., Kishimoto T. (1990). Molecular cloning and expression of an IL-6 signal transducer, gp130. Cell.

[63] Heinrich P.C., Behrmann I., Müller-Newen G., Schaper F., Graeve L. (1998). IL-6-type cytokine signalling through the gp130/JAK/STAT pathway. Biochem. J.

[64] Senaldi G., Varnum B.C., Sarmiento U., Starnes C., Lile J., Scully S., Guo J., Elliott G., McNinch J., Shaklee C.L. (1999). Novel neurotrophin-1/B cell-stimulating factor-3: A cytokine of the IL-6 family. Proc. Natl. Acad. Sci. USA.

[65] Davis S., Aldrich T.H., Valenzuela D.M., Wong V.V., Furth M.E., Squinto S.P., Yancopoulos G.D. (1991). The receptor forciliary neurotrophic factor. Science.

[66] Davis S., Yancopoulos G.D. (1993). The molecular biology of the CNTF receptor. Curr. Opin. Nelurobiol.

[67] Novick D., Engelmann H., Wallach D., Rubinstein M. (1989). Soluble cytokine receptors are present in normal human urine. J. Exp. Med.

[68] Honda M., Yamamoto S., Cheng M., Yasukawa K., Suzuki H., Saito T., Osugi Y., Tokunaga T., Kishimoto T. (1992). Human soluble IL-6 receptor: Its detection and enhanced release by HIV infection. J. Immunol.

[69] Mackiewicz A., Schooltink H., Heinrich P.C., Rose-John S. (1992). Complex of soluble human IL-6-receptor/IL-6 up-regulates expression of acute-phase proteins. J. Immunol.

[70] Taga T., Hibi M., Hirata Y., Yamasaki K., Yasukawa K., Matsuda T., Hirano T., Kishimoto T. (1989). Interleukin-6 triggers the association of its receptor with a possible signal transducer, gp130. Cell.

[71] Jones S.A., Richards P.J., Scheller J., Rose-John S. (2005). IL-6 transsignaling: The *in vivo* consequences. J. Interf. Cytokine Res.

[72] Rose-John S. (2002). GP130 stimulation and the maintenance of stem cells. Trends Biotechnol.

[73] Humphrey R.K., Beattie G.M., Lopez A.D., Bucay N., King C.C., Firpo M., Rose-John S., Hayek A. (2004). Maintenance of pluripotency in human embryonic stem cells is Stat3 independent. Stem. Cells.

[74] Peters M., Schirmacher P., Goldschmitt J., Odenthal M., Peschel C., Dienes H.P., Fattori E., Ciliberto G., Meyer zum Büschenfelde K.H., Rose-John S. (1997). Extramedullary expansion of hematopoietic progenitor cells in IL-6/sIL-6R double transgenic mice. J. Exp. Med.

[75] Peters M., Müller A., Rose-John S. (1998). Interleukin-6 and soluble interleukin-6 receptor: Direct stimulation of gp130 and hematopoiesis. Blood.

[76] Audet J., Miller C.L., Rose-John S., Piret J.M., Eaves C.J. (2001). Distinct role of gp130 activation in promoting self-renewal divisions by mitogenically stimulated murine hematopoietic cells. Proc. Natl. Acad. Sci. USA.

[77] Hacker C., Kirsch R.D., Ju X.-S., Hieronymus T., Gust T.C., Kuhl C., Jorgas T., Kurz S.M., Rose-John S., Yokota Y. (2003). Transcriptional profiling identifies Id2 function in dendritic cell development. Nat. Immunol.

[78] Campard D., Vasse M., Rose-John S., Poyer F., Lamacz M., Vannier J.P. (2005). Multilevel regulation of IL-6R by IL-6/sIL-6R fusion protein according to the primitiveness of the peripheral blood-derived CD133+ cells. Stem Cells.

[79] Atreya R., Mudter J., Finotto S., Müllberg J., Jostock T., Wirtz S., Schütz M., Bartsch B., Holtmann M., Becker C. (2000). Blockade of IL-6 transsignaling abrogates established experimental colitis in mice by suppression of the antiapoptotic resistance of lamina propria T cells. Nat. Med.

[80] Becker C., Fantini M.C., Schramm C., Lehr H.A., Wirtz S., Nikolaev A., Burg J., Strand S., Kiesslich R., Huber S. (2004). TGF-β suppresses tumor progression in colon cancer by inhibition of IL-6 trans-signaling. Immunity.

[81] Becker C., Fantini M.C., Wirtz S., Nikolaev A., Lehr H.A., Galle P.R., Rose-John S., Neurath M.F. (2005). IL-6 signaling promotes tumor growth in colorectal cancer. Cell Cycle.

[82] März P., Cheng J.-C., Gadient R.A., Patterson P., Stoyan T., Otten U., Rose-John S. (1998). Sympathetic neurons can produce and respond to interleukin-6. Proc. Natl. Acad. Sci. USA.

[83] März P., Otten U., Rose-John S. (1999). Neuronal activities of IL-6 type cytokines often depend on soluble cytokine receptors. Eur. J. Neurosci.

[84] Klouche M., Bhakdi S., Hemmes M., Rose-John S. (1999). Novel path of activation of primary human smooth muscle cells: Upregulation of gp130 creates an autocrine activation loop by IL-6 and its soluble receptor. J. Immunol.

[85] Hurst S.M., Wilkinson T.S., McLoughlin R.M., Jones S., Horiuchi S., Yamamoto N., Rose-John S., Fuller G.M., Topley N., Jones S.A. (2001). Control of leukocyte infiltration during inflammation: IL-6 and its soluble receptor orchestrate a temporal switch in the pattern of leukocyte recruitment. Immunity.

[86] Mcloughlin R.M., Witowski J., Robson R.L., Wilkinson T.S., Hurst S.M., Williams A.S., Williams J.D., Rose-John S., Jones S.A., Topley N. (2003). Interplay between IFN-γ and IL-6 signaling governs neutrophil trafficking and apoptosis during acute inflammation. J. Clin. Invest.

[87] Romano M., Sironi M., Toniatti C., Polentarutti N., Fruscella P., Ghezzi P., Faggioni R., Luini W., van Hinsbergh V., Sozzani S. (1997). Role of IL-6 and its soluble receptor in induction of chemokines and leukocyte recruitment. Immunity.

[88] Mendoçna Torres P.M., de Araujo E.G. (2001). Interleukin-6 increases the survival of retinal ganglion cells *in vitro*. J. Neuroimmunol.

[89] Hirota H., Kiyama H., Kishimoto T., Taga T. (1996). Accelerated nerve regeneration in mice by upregulated expression of interleukin-6 (IL-6) and IL-6 receptor after trauma. J. Exp. Med.

[90] Haggiag S., Chebath J., Revel M. (1999). Induction of myelin gene expression in Schwann cell cultures by an interleukin-6 receptor-interleukin-6 chimera. FEBS Lett.

[91] Brunello A.G., Weissenberger J., Kappeler A., Vallan C., Peters M., Rose-John S., Weis J. (2000). Astrocytic alterations in interleukin-6/soluble interleukin-6 receptor α double transgenic mice. Am. J. Pathol.

[92] Yang X.J., Chung D., Cepko C.L. (1993). Molecular cloning of the murine JAK1 protein tyrosine kinase and its expression in the mouse central nervous system. J. Neurosci.

[93] De-Fraja C., Conti L., Magrassi L., Govoni S., Cattaneo E. (1998). Members of the JAK/STAT proteins are expressed and regulated during development in the mammalian forebrain. J. Neurosci. Res..

[94] Darnell J.E., Kerr I.M., Stark G.R. (1994). Jak-STAT pathways and transcriptional activation in response to IFNs and other extracellular proteins. Science.

[95] Akira S., Nishio Y., Inoue M., Wang X., We S., Matsusaka T., Yoshida K., Sudo T., Naruto M., Kishimot T. (1994). Molecular cloning of APRF, a novel IFN-stimulated gene factor 3 p91-related transcription factor involved in the gp130-mediated signaling pathway. Cell.

[96] Lütticken C., Wegenka U.M., Yuan J., Buschmann J., Schindler C., Ziemiecki A., Harpu A.G., Wilks A.F., Yasukawa K., Taga T. (1994). Association of transcription factor APRF and protein kinase Jak1 with the interleukin-6 signal transducer gp130. Science.

[97] Zhong Z., Wen Z., Darnell J.E. (1994). Stat3: A STAT family member activated by tyrosine phosphorylation in response to epidermal growth factor and interleukin-6. Science.

[98] Raz R., Durbin J.E., Levy D.E. (1994). Acute phase response factor and additional members of the interferon-stimulated gene factor 3 family integrate diverse signals from cytokines, interferons, and growth factors. J. Biol. Chem.

[99] Hirano T., Ishihara K., Hibi M. (2000). Roles of STAT3 in mediating the cell growth, differentiation and survival signals relayed through the IL-6 family of cytokine receptors. Oncogene.

[100] März P., Herget T., Lang E., Otten U., Rose-John S. (1998). Activation of gp130 by IL-6/soluble IL-6 receptor induces neuronal differentiation. Eur. J. Neurosci.

[101] Boeuf H., Hauss C., Graeve F.D., Baran N., Kedinger C. (1997). Leukemia inhibitory factor-dependent transcriptional activation in embryonic stem cells. J. Cell Biol.

[102] Niwa H., Burdon T., Chambers I., Smith A. (1998). Self-renewal of pluripotent embryonic stem cells is mediated via activation of STAT3. Genes Dev.

[103] Raz R., Lee C.K., Cannizzaro L.A., d’Eustachio P., Levy D.E. (1999). Essential role of STAT3 for embryonic stem cell pluripotency. Proc Natl. Acad. Sci. USA.

[104] Matsuda T., Nakamura T., Nakao K., Arai T., Katsuki M., Heike T., Yokota T. (1999). STAT3 activation is sufficient to maintain an undifferentiated state of mouse embryonic stem cells. EMBO. J.

[105] Bonni A., Sun Y., Nadal-Vicens M., Bhatt A., Frank D.A., Rozovsky I., Stahl N., Yancopoulos G.D., Greenberg M.E. (1997). Regulation of gliogenesis in the central nervous system by the JAK-STAT signaling pathway. Science.

[106] Gu F., Hata R., Ma Y.J., Tanaka J., Mitsuda N., Kumon Y., Hanakawa Y., Hashimoto K., Nakajima K., Sakanaka M. (2005). Suppression of Stat3 promotes neurogenesis in cultured neural stem cells. J. Neurosci. Res..

[107] Yanagisawa M., Nakashima K., Taga T. (1999). STAT3-mediated astrocyte differentiation from mouse fetal neuroepithelial cells by mouse oncostatin M. Neurosci. Lett.

[108] Fukuda S., Abematsu M., Mori H., Yanagisawa M., Kagawa T., Nakashima K., Yoshimura A., Taga T. (2007). Potentiation of astrogliogenesis by STAT3-mediated activation of bone morphogenetic protein-Smad signaling in neural stem cells. Mol. Cell Biol.

[109] Taylor M.K., Yeager K., Morrison S.J. (2007). Physiological Notch signaling promotes gliogenesis in the developing peripheral and central nervous systems. Development.

[110] Takeda K., Noguchi K., Shi W., Tanaka T., Matsumoto M., Yoshida N., Kishimoto T., Akira S. (1997). Targeted disruption of the mouse Stat3 gene leads to early embryonic lethality. Proc. Natl. Acad. Sci. USA.

[111] Yoshida K., Taga T., Saito M., Suematsu S., Kumanogoh A., Tanaka T., Fujiwara H., Hirata M., Yamagami T., Nakahata T. (1996). Targeted disruption of gp130, a common signal transducer for the interleukin 6 family of cytokines, leads to myocardial and hematological disorders. Proc. Natl. Acad. Sci. USA.

[112] Guschin D., Rogers N., Briscoe J., Witthuhn B., Watling D., Horn F., Pellegrini S., Yasukawa K., Heinrich P., Stark G.R. (1995). A major role for the protein tyrosine kinase JAK1 in the JAK/STAT signal transduction pathway in response to interleukin-6. EMBO. J.

[113] Ernst M., Oates A., Dunn A.R. (1996). Gp130-mediated signal transduction in embryonic stem cells involves activation of Jak and Ras/mitogen-activated protein kinase pathways. J. Biol. Chem.

[114] Rodig S.J., Meraz M.A., White J.M., Lampe P.A., Riley J.K., Arthur C.D., King K.L., Sheehan K.C., Yin L., Pennica D. (1998). Disruption of the Jak1 gene demonstrates obligatory and nonredundant roles of the Jaks in cytokine-induced biologic responses. Cell.

[115] Moon C., Yoo J.Y., Matarazzo V., Sung Y.K., Kim E.J., Ronnett G.V. (2002). Leukemia inhibitory factor inhibits neuronal terminal differentiation through STAT3 activation. Proc. Natl. Acad. Sci. USA.

[116] Pan J., Fukuda K., Kodama H., Sano M., Takahashi T., Makino S., Kato T., Manabe T., Hori S., Ogawa S. (1998). Involvement of gp130-mediated signaling in pressure overload-induced activation of the JAK/STAT pathway in rodent heart. Heart Vessel..

[117] Wu Y.Y., Bradshaw R.A. (1996). Induction of neurite outgrowth by interleukin-6 is accompanied by activation of Stat3 signaling pathway in a variant PC12 cell (E2) line. J. Biol. Chem.

[118] Wu Y.Y., Bradshaw R.A. (1996). Synergistic induction of neurite outgrowth by nerve growth factor or epidermal growth factor and interleukin-6 in PC12 cells. J. Biol. Chem.

[119] Ihara S., Nakajima K., Fukada T., Hibi M., Nagata S., Hirano T., Fukui Y. (1997). Dual control of neurite outgrowth by STAT3 and MAP kinase in PC12 cells stimulated with interleukin-6. EMBO J..

[120] Rajan P., McKay R.D.G. (1998). Multiple routes to astrocytic differentiation in the CNS. J. Neurosci.

[121] Rajan P., Symes A.J., Fink J.S. (1996). Stat proteins are activated by ciliary neurotrophic factor in cells of central nervous system origin. J. Neurosci. Res.

[122] Symes A., Lewis S., Corpus L., Rajan P., Hyman S.E., Fink J.S. (1994). STAT proteins participate in the regulation of the vasoactive intestinal peptide gene by the ciliary neurotrophic factor family of cytokines. Mol. Endocrinol.

[123] Hamanaka I., Saito Y., Yasukawa H., Kishimoto I., Kuwahara K., Miyamoto Y., Harada M., Ogawa E., Kajiyama N., Takahashi N., Izumi T. (2001). Induction of JAB/SOCS-1/SSI-1 and CIS3/SOCS-3/SSI-3 is involved in gp130 resistance in cardiovascular system in rat treated with cardiotrophin-1 *in vivo*. Circ. Res.

[124] Stephanou A., Brar B.K., Knight R.A., Latchman D.S. (2000). Opposing actions of STAT-1 and STAT-3 on the Bcl-2 and Bcl-x promoters. Cell Death Differ.

[125] Sheng Z., Knowlton K., Chen J., Hoshijima M., Brown J.H., Chien K.R. (1997). Cardiotrophin 1 (CT-1) inhibition of cardiac myocyte apoptosis via a mitogen-activated protein kinase-dependent pathway. Divergence from downstream CT-1 signals for myocardial cell hypertrophy. J. Biol. Chem.

[126] Shi Y., Wang W., Yourey P.A., Gohari S., Zukauskas D., Zhang J., Ruben S., Alderson R.F. (1999). Computational EST database analysis identifies a novel member of the neuropoietic cytokine family. Biochem. Biophys. Res. Commun.

[127] Auernhammer C.J., Isele N.B., Kopp F.B., Spoettl G., Cengic N., Weber M.M., Senaldi G., Engelhardt D. (2003). Novel neurotrophin-1/B cell-stimulating factor-3 (cardiotrophin-like cytokine) stimulates corticotroph function via a signal transducer and activator of transcription-dependent mechanism negatively regulated by suppressor of cytokine signaling-3. Endocrinology.

[128] Boulton T.G., Stahl N., Yancopoulos G.D. (1994). Ciliary neurotrophic factor/leukemia inhibitory factor/interleukin 6/oncostatin M family of cytokines induces tyrosine phosphorylation of a common set of proteins overlapping those induced by other cytokines and growth factors. J. Biol. Chem..

[129] Guzzo C., Fazila N., Mat C., Gee K. (2012). Interleukin-27 Induces a STAT1/3- and NF-κB-dependent proinflammatory cytokine profile in human monocytes. J. Biol. Chem.

[130] Dambacher J., Beigel F., Seiderer J., Haller D., Göke B., Auernhammer C.J., Brand S. (2007). Interleukin 31 mediates MAP kinase and STAT1/3 activation in intestinal epithelial cells and its expression is upregulated in inflammatory bowel disease. Gut.

[131] Zigmond R.E. (2011). gp130 cytokines are positive signals triggering changes in gene expression and axon outgrowth in peripheral neurons following injury. Front. Mol. Neurosci.

[132] Sun F., He Z. (2010). Neuronal intrinsic barriers for axon regeneration in the adult CNS. Curr. Opin. Neurobiol.

[133] Pflanz S., Hibbert L., Mattson J., Rosales R., Vaisberg E., Bazan J.F., Phillips J.H., McClanahan T.K., de Waal Malefyt R., Kastelein R.A. (2004). WSX-1 and glycoprotein 130 constitute a signal-transducing receptor for IL-27. J. Immunol.

[134] Levy D.E., Danell J.E. (2002). Stats: Transcriptional control and biological impact. Nat. Rev. Mol. Cell Biol..

[135] Brivanlou A.H., Darnell J.E. (2002). Signal transduction and the control of gene expression. Science.

[136] O’Shea J.J., Gadina M., Schreiber R.D. (2002). Cytokine signaling in 2002: New surprises in the Jak/Stat pathway. Cell.

[137] Guo Z., Jiang H., Xu X., Duan W., Mattson M.P. (2008). Leptin-mediated cell survival signaling in hippocampal neurons mediated by JAK STAT3 and mitochondrial stabilization. J. Biol. Chem.

[138] Repovic P., Benveniste E.N. (2002). Prostaglandin E2 is a novel inducer of oncostatin-M expression in macrophages and microglia. J. Neurosci.

[139] Van Wagoner N.J., Benveniste E.N. (1999). Interleukin-6 expression and regulation in astrocytes. J. Neuroimmunol.

[140] Sun Y., Marz P., Otten U., Ge J., Rose-John S. (2002). The effect of gp130 stimulation on glutamate-induced excitotoxicity in primary hippocampal neurons. Biochem. Biophys. Res. Commun.

[141] Hatta T., Moriyama K., Nakashima K., Taga T., Otani H. (2002). The role of gp130 in cerebral cortical development: *In vivo* functional analysis in a mouse exo utero system. J. Neurosci.

[142] Fukada T., Hibi M., Yamanaka Y., Takahashi-Te zuka M., Fujitani Y., Yamaguchi T., Nakajima K., Hirano T. (1996). Two signals are necessary for cell proliferation induced by a cytokine receptor gp130: Involvement of STAT3 in anti-apoptosis. Immunity.

[143] Takeda K., Kaisho T., Yoshida N., Takeda J., Kishimoto T., Akira S. (1998). Stat3 activation is responsible for IL-6-dependent T cell proliferation through preventing apoptosis: Generation and characterization of T cell-specific Stat3-deficient mice. J. Immunol.

[144] Catlett-Falcone R., Landowski T.H., Oshiro M.M., Turkson J., Levitzki A., Savino R., Ciliberto G., Moscinski L., Fernandez-Luna J.L., Nunez G. (1999). Constitutive activation of Stat3 signaling confers resistance to apoptosis in human U266 myeloma cells. Immunity.

[145] Scheller J., Grötzinger J., Rose-John S. (2006). Updating IL-6 classic- and trans-signaling. Signal Transduct.

[146] Müllberg J., Geib T., Jostock T., Hoischen S.H., Vollmer P., Voltz N., Heinz D., Galle P.R., Klouche M., Rose-John S. (2000). IL-6-receptor independent stimulation of human gp130 by viral IL-6. J. Immunol.

[147] Pennica D., Wood W.I., Chien K.R. (1996). Cardiotrophin-1: A multifunctional cytokine that signals via LIF receptor-gp 130 dependent pathways. Cytokine Growth Factor Rev.

[148] Turnley A.M., Bartlett P.F. (2000). Cytokines that signal through the leukemia inhibitory factor receptor-b complex in the nervous system. J. Neurochem.

[149] Murphy M., Dutton R., Koblar S., Cheema S., Bartlett P. (1997). Cytokines which signal through the LIF receptor and their actions in the nervous system. Prog. Neurobiol.

[150] Patterson P.H. (1994). Leukemia inhibitory factor, a cytokine at the interface between neurobiology and immunology. Proc. Natl. Acad. Sci. USA.

[151] Anneren C. (2008). Tyrosine kinase signalling in embryonic stem cells. Clin. Sci.

[152] Miao T., Wu D., Zhang Y., Bo X., Subang M.C., Wang P., Richardson P.M. (2006). Suppressor of cytokine signaling-3 suppresses the ability of activated signal transducer and activator of transcription-3 to stimulate neurite growth in rat primary sensory neurons. J. Neurosci.

[153] Bareyre F.M., Garzorz N., Lang C., Misgeld T., Buning H., Kerschensteiner M. (2011). *In vivo* imaging reveals a phase-specific role of STAT3 during central and peripheral nervous system axon regeneration. Proc. Natl. Acad. Sci. USA.

[154] Boulanger M.J., Chow D.C., Brevnova E.E., Garcia K.C. (2003). Hexameric structure and assembly of the interleukin-6/IL-6 alpha-receptor/gp130 complex. Science.

[155] Lee J.C., Mayer-Proschel M., Rao M.S. (2000). Gliogenesis in the central nervous system. Glia.

[156] Bauer S., Kerr B.J., Patterson P.H. (2007). The neuropoietic cytokine family in development plasticity, disease and injury. Nat. Rev. Neurosci..

[157] Thier M., Hall M., Heath J.K., Pennica D., Weis J. (1999). Trophic effects of cardiotrophin-1 and interleukin-11 on rat dorsal root ganglion neurons *in vitro*. Brain Res. Mol. Brain Res.

[158] De Melo Reis R.A., Schitine C.S., Köfalvi A., Grade S., Cortes L., Gardino P.F., Malva J.O., de Mello F.G. (2011). Functional identification of cell phenotypes differentiating from mice retinal neurospheres using single cell calcium imaging. Cell Mol. Neurobiol.

[159] Homs J., Ariza L., Pagès G., Udina E., Navarro X., Chillón M., Bosch A. (2011). Schwann cell targeting via intrasciatic injection of AAV8 as gene therapy strategy for peripheral nerve regeneration. Gene Ther.

[160] Bauer S. (2009). Cytokine control of adult neural stem cells. Ann. N. Y. Acad. Sci..

[161] Scheller J., Chalaris A., Schmidt-Arras D., Rose-John S. (2011). The pro- and anti-inflammatory properties of the cytokine interleukin-6. Biochim. Biophys. Acta.

[162] Gadient R.A., Otten U. (1994). Expression of interleukin-6 (IL-6) and interleukin-6 receptor (IL-6R) mRNAs in rat brain during postnatal development. Brain Res.

[163] Wagner J.A. (1996). Is IL-6 both a cytokine and a neurotrophic factor?. J. Exp. Med.

[164] Monje M.L., Toda H., Palmer T.D. (2003). Inflammatory blockade restores adult hippocampal neurogenesis. Science.

[165] Nakanishi M., Niidome T., Matsuda S., Akaike A., Kihara T., Sugimoto H. (2007). Microglia-derived interleukin-6 and leukaemia inhibitory factor promote astrocytic differentiation of neural stem/progenitor cells. Eur. J. Neurosci.

[166] Akaneya Y., Takahashi M., Hatanaka H. (1995). Interleukin-1 beta enhances survival and interleukin-6 protects against MPP + neurotoxicity in cultures of fetal rat dopaminergic neurons. Exp. Neurol.

[167] Hama T., Miyamoto M., Tsukui H., Nishio C., Hatanaka H. (1989). Interleukin-6 as a neurotrophic factor for promoting the survival of cultured basal forebrain cholinergic neurons from postnatal rats. Neurosci. Lett.

[168] Hama T., Kushima Y., Miyamoto M., Kubota M., Takei N., Hatanaka H. (1991). Interleukin-6 improves the survival of mesencephalic catecholaminergic and septal cholinergic neurons from postnatal, two-week-old rats in cultures. Neuroscience.

[169] Kushima Y., Hama T., Hatanaka H. (1992). Interleukin-6 as a neurotrophic factor for promoting the survival of cultured catecholaminergic neurons in a chemically defined medium from fetal and postnatal rat midbrains. Neurosci. Res.

[170] Kushima Y., Hatanaka H. (1992). Interleukin-6 and leukemia inhibitory factor promote the survival of acetylcholinesterase-positive neurons in culture from embryonic rat spinal cord. Neurosci. Lett.

[171] Loddick S.A., Turnbull A.V., Rothwell N.J. (1998). Cerebral interleukin-6 is neuroprotective during permanent focal cerebral ischemia in the rat. J. Cereb. Blood Flow Metab.

[172] Matsuda S., Wen T.C., Morita F., Otsuka H., Igase K., Yoshimura H., Sakanaka M. (1996). Interleukin-6 prevents ischemia-induced learning disability and neuronal and synaptic loss in gerbils. Neurosci. Lett..

[173] Toulmond S., Vige X., Fage D., Benavides J. (1992). Local infusion of interleukin-6 attenuates the neurotoxic effects of NMDA on rat striatal cholinergic neurons. Neurosci. Lett.

[174] Yamada M., Hatanaka H. (1994). Interleukin-6 protects cultured rat hippocampal neurons against glutamate-induced cell death. Brain Res.

[175] Gadient R.A., Otten U.H. (1997). Interleukin-6 (IL-6)—A molecule with both beneficial and destructive potentials. Prog. Neurobiol.

[176] Ihara S., Iwamatsu A., Fujiyoshi T., Komi A., Yamori T., Fukui Y. (1996). Identification of interleukin-6 as a factor that induces neurite outgrowth by PC12 cells primed with NGF. J. Biochem.

[177] Boulanger L.M. (2009). Immune proteins in brain development and synaptic plasticity. Neuron.

[178] Pickering M., O’Connor J.J. (2007). Pro-inflammatory cytokines and their effects in the dentate gyrus. Prog. Brain Res.

[179] Vitkovic L., Konsman J.P., Bockaert J., Dantzer R., Homburger V., Jacque C. (2000). Cytokine signals propagate through the brain. Mol. Psychiatry.

[180] Viviani B., Gardoni F., Marinovich M. (2007). Cytokines and neuronal ion channels in health and disease. Int. Rev. Neurobiol.

[181] Kahn M.A., De Vellis J. (1994). Regulation of an oligodendrocyte progenitor cell line by the interleukin-6 family of cytokines. Glia.

[182] Valerio A., Ferrario M., Dreano M., Garotta G., Spano P., Pizzi M. (2002). Soluble interleukin-6 (IL-6) receptor/IL-6 fusion protein enhances *in vitro* differentiation of purified rat oligodendroglial lineage cells. Mol. Cell Neurosci.

[183] Zhang P.L., Izrael M., Ainbinder E., Ben-Simchon L., Chebath J., Revel M. (2006). Increased myelinating capacity of embryonic stem cell derived oligodendrocyte precursors after treatment by interleukin-6/soluble interleukin-6 receptor fusion protein. Mol. Cell Neurosci.

[184] Islam O., Gong X., Rose-John S., Heese K. (2009). Interleukin-6 and neural stem cells: More than gliogenesis. Mol. Biol. Cell.

[185] Oh J., McCloskey M.A., Blong C.C., Bendickson L., Nilsen-Hamilton M., Sakaguchi D.S. (2010). Astrocyte-derived interleukin-6 promotes specific neuronal differentiation of neural progenitor cells from adult hippocampus. J. Neurosci. Res.

[186] Yang Y.C., Yin T. (1992). Inteleukin-11 and its receptor. Biofiictors.

[187] Du X.X., Williams D.A. (1994). Interleukin-11: A multifunctional growth factor derived from the hematopoietic microenvironment. Blood.

[188] Paul S.R., Bennett F., Calvetti J.A., Kelleher K., Wood C.R., OHara R.M., Leary A.C. (1990). Molecular cloning of a cDNA encoding interleukin 11, a stromal cell-derived lymphopoietic and hematopoietic cytokine. Proc. Natl. Acad. Sci. USA.

[189] Rose T.M., Bruce A.G. (1991). Oncostatin M is a member of a cytokine family that includes leukemia-inhibitory factor, granulocyte colony-stimulating factor, and interleukin 6. Proc. Natl. Acad. Sci. USA.

[190] Hilton D.J. (1992). LIF: Lots of interesting functions. Trends Biochem. Sci.

[191] Kishimoto T., Akira S., Taga T. (1992). Interleukin-6 and its receptor: A paradigm for cytokines. Science.

[192] Davis S., Aldrich T.H., Stahl N., Pan L., Taga T., Kishimoto T., Ip N.Y., Yancopolous G.D. (1993). LIFR beta and gp130 as heterodimerizing signal transducers of the tripartite CNTF receptor. Science.

[193] Chang M.Y., Park C.H., Son H., Lee Y.S., Lee S.H. (2004). Developmental stage-dependent self-regulation of embryonic cortical precursor cell survival and differentiation by leukemia inhibitory factor. Cell Death Differ..

[194] Dowsing B.J., Hayes A., Bennett T.M., Morrison W.A., Messina A. (2000). Effects of LIF dose and laminin plus fibronectin on axotomized sciatic nerves. Muscle Nerve.

[195] Cheema S.S., Richards L., Murphy M., Bartlett P.F. (1994). Leukemia inhibitory factor prevents the death of axotomised sensory neurons in the dorsal root ganglia of the neonatal rat. J. Neurosci. Res.

[196] Ikeda K., Iwasaki Y., Shiojima T., Kinoshita M. (1996). Neuroprotective effect of various cytokines on developing spinal motoneurons following axotomy. J. Neurol. Sci.

[197] Ikeda K., Iwasaki Y., Tagaya N., Shiojima T., Kinoshita M. (1995). Neuroprotective effect of cholinergic differentiation factor/leukemia inhibitory factor on wobbler murine motor neuron disease. Muscle Nerve.

[198] Azari M.F., Galle A., Lopes E.C., Kurek J., Cheema S.S. (2001). Leukemia inhibitory factor by systemic administration rescues spinal motor neurons in the SOD1 G93A murine model of familial amyotrophic lateral sclerosis. Brain Res.

[199] Bauer S., Patterson P.H. (2006). Leukemia inhibitory factor promotes neural stem cell self renewal in the adult brain. J. Neurosci.

[200] Majumder A., Banerjee S., Harrill J.A., Machacek D.W., Mohamad O., Bacanamwo M., Mundy W.R., Wei L., Dhara S.K., Stice S.L. (2012). Neurotrophic effects of leukemia inhibitory factor on neural cells derived from human embryonic stem cells. Stem Cells.

[201] Galli R., Pagano S.F., Gritti A., Vescovi A.L. (2000). Regulation of neuronal differentiation in human CNS stem cell progeny by leukemia inhibitory factor. Dev. Neurosci.

[202] Buono K.D., Vadlamuri D., Gan Q., Levison S.W. (2012). Leukemia inhibitory factor is essential for subventricular zone neural stem cell and progenitor homeostasis as revealed by a novel flow cytometric analysis. Dev. Neurosci.

[203] Carpenter M.K., Cui X., Hu Z.Y., Jackson J., Sherman S., Seiger A., Wahlberg L.U. (1999). *In vitro* expansion of a multipotent population of human neural progenitor cells. Exp. Neurol.

[204] Wright L.S., Li J., Caldwell M.A., Wallace K., Johnson J.A., Svendsen C.N. (2003). Gene expression in human neural stem cells: Effects of leukemia inhibitory factor. J. Neurochem.

[205] Pagano S.F., Impagnatiello F., Girelli M., Cova L., Grioni E., Onofri M., Cavallaro M., Etteri S., Vitello F., Giombini S. (2000). Isolation and characterization of neural stem cells from the adult human olfactory bulb. Stem Cells.

[206] Barnabe-Heider F., Wasylnka J.A., Fernandes K.J., Porsche C., Sendtner M., Kaplan D.R., Miller F.D. (2005). Evidence that embryonic neurons regulate the onset of cortical gliogenesis via cardiotrophin-1. Neuron.

[207] Ishibashi T., Dakin K.A., Stevens B., Lee P.R., Kozlov S.V., Stewart C.L., Fields R.D. (2006). Astrocytes promote myelination in response to electrical impulses. Neuron.

[208] Schmiz T., Chew L.J. (2008). Cytokines and myelination in the central nervous system. Sci. World J.

[209] Mitsumoto H., Ikeda K., Klinkosz B., Cedarbaum J.M., Wong V., Lindsay R.M. (1994). Arrest of motor neuron disease in wobbler mice cotreated with BDNF and CNTF. Science.

[210] Leibinger M., Müller A., Andreadaki A., Hauk T.G., Kirsch M., Fischer D. (2009). Neuroprotective and axon growth-promoting effects following inflammatory stimulation on mature retinal ganglion cells in mice depend on ciliaryneurotrophic factor and leukemia inhibitory factor. J. Neurosci.

[211] Butzkueven H., Emery B., Cipriani T., Marriott M.P., Kilpatrick T.J. (2006). Endogenous leukemia inhibitory factor production limits autoimmune demyelination and oligodendrocyte loss. Glia.

[212] White U.A., Stephens J.M. (2010). Neuropoietin activates STAT3 independent of LIFR activation in adipocytes. Biochem. Biophys. Res. Commun.

